# A geometric design method for intersections with pre-signal systems using a phase swap sorting strategy

**DOI:** 10.1371/journal.pone.0217741

**Published:** 2019-05-31

**Authors:** Yan Li, Sirui Nan, Xiaolin Gong, Rui Ma

**Affiliations:** 1 School of Highway, Chang’an University, Xi’an, Shaanxi, China; 2 CCCC First Highway Consultants Co., LTD, Xi’an, Shaanxi, China; 3 Department of Civil, Geo and Environmental Engineering, Technical University of Munich, Munich, Bavaria, Germany; 4 Department of Civil and Environmental Engineering, University of California, Davis, Davis, California, United States of America; Huazhong University of Science and Technology, CHINA

## Abstract

Conventional geometric design methods for pre-signal systems usually use the expected traffic demand, which may obtain a short sorting area distance and lead to frequent queue spillbacks due to stochastic traffic arrivals. On the other hand, if one selects a longer sorting area distance, the geometric design will suffer from low spatial utilization with higher delay and lower capacity. In this paper, we propose a geometric design method for intersections with pre-signal systems using a phase swap strategy. The geometric design can balance the desire of storing more vehicles to prevent spillbacks and improve the spatial utilization of the road. We model the traffic dynamic within the pre-signal system using queue theory and shockwave theory to determine the furthest point a queue can reach. The length of the pre-signal system should be short enough to improve spatial utilization but longer than the furthest point of the queue to prevent queue spillback. The effectiveness of the pre-signal system is evaluated by the VISSIM Signal Control Application Programming Interfaces (SCAPI). The results indicate that the proposed design plan increases the spatial utilization of the pre-signal system by 7.5% while maintaining a similar delay, queue length and ratio of flow to saturation flow.

## Introduction

### Background

Conventional traffic signal control improves the efficiency of at-grade intersections by separating conflicting movements in time[1]. When the traffic demand approaches the road capacity, conventional traffic signal control cannot significantly improve traffic efficiency[2]. One of the possible ways to improve traffic efficiency is to increase the capacity for bottleneck movement[3]. Because the road space in modern cities is often limited, commonly used methods, such as increasing the number of lanes or setting the turning bay[4], are difficult to implement. The pre-signal system[5], which adds an auxiliary traffic signal group at the upstream section of the intersection approach, makes it possible to use all the road space between two stop lines for one movement at a specific time interval, without requiring additional road space. The positioning of the pre-signal system can increase the effective number of lanes for discharging vehicles or, equivalently, allow more room for the optimization of traffic signal timing, which can significantly improve the traffic efficiency under congestion status. However, the pre-signal system increases the complexity of traffic management and control of the intersection. Inappropriate geometric design and uncoordinated signal timings between the pre-signal and main signal will be the most critical causes of the low operational efficiency. If the pre-signal system is “switched on” under the congestion status, the signal timing plan and geometric layout need to be carefully designed to keep all the vehicles running orderly and efficiently.

**Fig 1:**
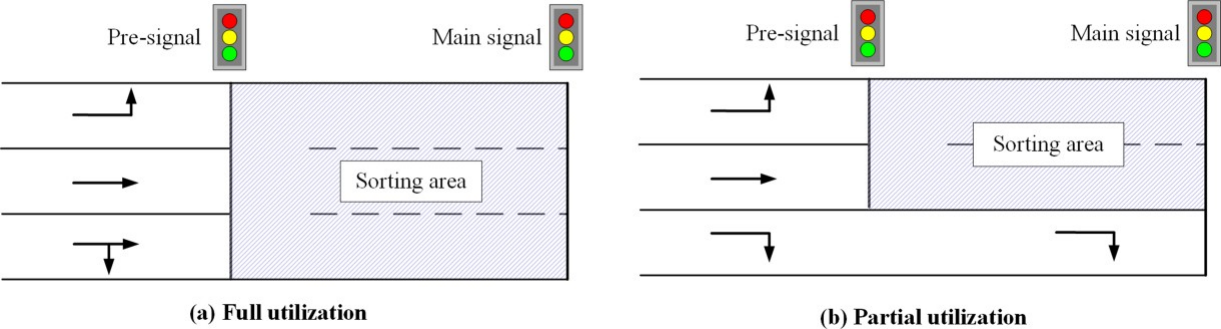
General layout of the pre-signal system.

The pre-signal re-organizes traffic in the sorting area using a tandem sorting or phase swap sorting traffic control strategy[6]. The major difference between the tandem sorting and phase swap sorting is the phase sequence. Compared with the tandem sorting strategy, the phase swap sorting can fill the sorting area during the green phase stage in the crossing direction[7]. Thus, the phase swap sorting can then be considered as an ideal method for the pre-signal due to its better time utilization. As shown in Fig 1, both tandem sorting and swap sorting will stop all traffic at the pre-signal stop line, and then tandemly discharges traffic heading the same direction into the sorting area. By filtering the traffic using the pre-signal, the main signal can use all the cross-sections of the sorting area to discharge vehicles heading to the same direction. In addition, all the phase stages of the main signal will still be protected. In short, left turning vehicles (LV) and throughput vehicles (TV) will form tandem batches in the pre-signal system and travel through the sorting area as well as the intersection cross-section using all the lanes.

As illustrated in Fig 1, the pre-signal system can consider all of the lanes as the sorting area[8], which can be termed full utilization, or can utilize two or more lanes for a specific turning movement or special usage, such as bus transit[9], which can be termed partial utilization. Once the lane allocation of the sorting area is selected, the parameters that need to be determined are the minimum length of the road segment located upstream of the pre-signal stop line and the length of the sorting area. Once the geometric design is implemented, it cannot be altered easily, which means the geometric design should be able to service most traffic conditions without reducing efficiency. Although the pre-signal can meter the vehicles entering the sorting area, the pre-signal system can easily experience queue spillbacks when the length of the sorting area or the upstream area is determined using the expected traffic demand. On the other hand, there is no queue spillback when the sorting area and upstream area are long enough. However, the spatial utilization of the road will drop significantly as the length increases. When the sorting area length is too long, it will cause an unnecessary stochastic delay. There will even be additional travel delay and capacity drop if the geometric design and related signal timing don’t match the variation of the saturated flow rate of the main signal. Thus, the geometric design of the pre-signal system should be carefully optimized to provide enough road space to park most vehicles and maintain high operational efficiency.

In this paper, a geometric design of the pre-signal system is determined that will improve operational efficiency and eliminate the queue spillbacks caused by stochastic traffic arrival or queue dispersion. We modeled the traffic dynamic of the sorting area and upstream area to locate the furthest point the queue can reach. The geometric design of the pre-signal system can then be determined by preventing queue spillback for most stochastically arriving traffic and improving the operational efficiency of the intersection with the pre-signal system.

### Literature review

The concept of a tandem intersection has developed from the bus pre-signal. Similar to the concept of the bike box[10], the bus pre-signal system provides a bus advance area between the main signal and bus pre-signal, in which buses can advance while the pre-signal is red and then be discharged ahead of private vehicles[11–13].

Theoretically, a pre-signal system for all vehicles can provide extra capacity for turning vehicles without reducing the capacity for throughput vehicles. The performance of the pre-signal system is affected by both the signal timing plan and the geometric design. The lane allocation, the length of the sorting area and the signal coordination between the pre-signal and the main signal all interact with each other. A suboptimal design may lead to heavy congestion[14]. Early models follow conventional signal timing methods. The performance indicators, such as delay and capacity, were estimated by using the probability model[5], Webster delay model[6] and shockwave model[15]. These models have little considerations on geometric designs. Newly developed design methods for the pre-signal system can optimize signal timing with the geometric design[7, 16]. These optimization problems were usually solved using Binary-Mixed-Integer-Linear-Programming (BMILP) model[17–18]. Because the “optimal” design calculated by BMILP model is based on the maximum queue length under expected traffic demand, this design is usually short. Although the signal timing plan of the pre-signal system can be determined in real time using the dynamic programming[19], the geometric design parameters are determined using the maximum vehicles can be discharged by the pre-signal, which is longer than the length needed. This method also needs to install enough detectors, which will increase the overall cost. Though many optimization models were developed to determine an appropriate design for the pre-signal system, these models are usually difficult to be directly applied due to the randomness of traffic demand and the physical environmental limitations of the intersections.

Numerical simulations indicate that a pre-signal system with an ideal configuration can increase the capacity of an intersection approach with three lanes by 15–50%[6, 15]. The operational efficiency of the intersection (such as delay or number of stops) with pre-signal is lower than the one of the conventional intersections when the saturation degree is less than 0.9. However, when the traffic demand approaches or exceeds the original capacity, the pre-signal system can maintain relatively high efficiency [16]. The reserved capacity of the pre-signal system can be as much as twice of the conventional design [7]. Based on field observations in Shenzhen, the capacity of the intersection increases by approximately 13%[14]. The principal reason for the difference between the observations and the theory is that drivers need additional knowledge to deal with the instructions given by the pre-signal[20–21].

**Fig 2:**
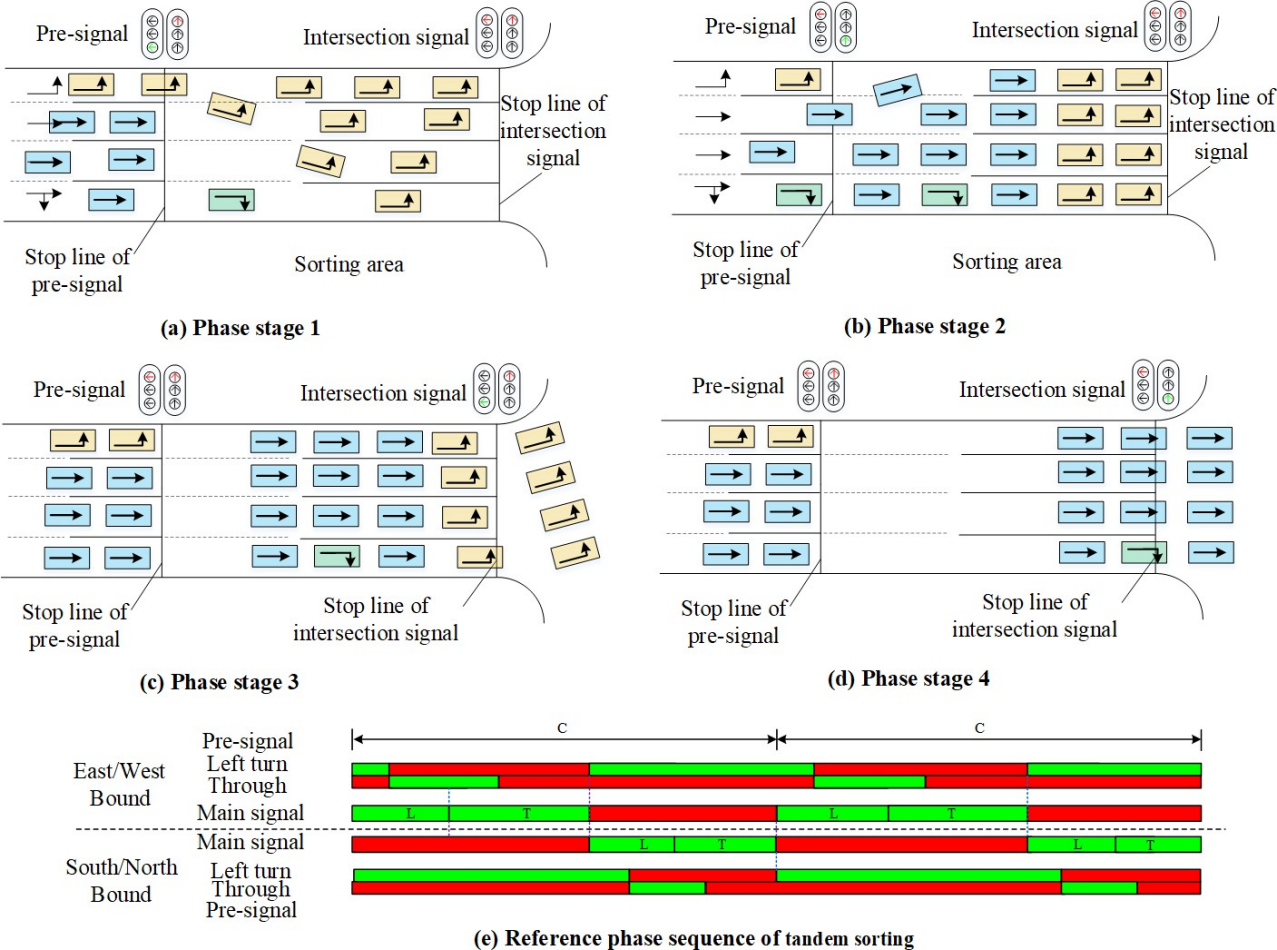
Components and operation of the pre-signal system with a tandem design.

**Fig 3:**
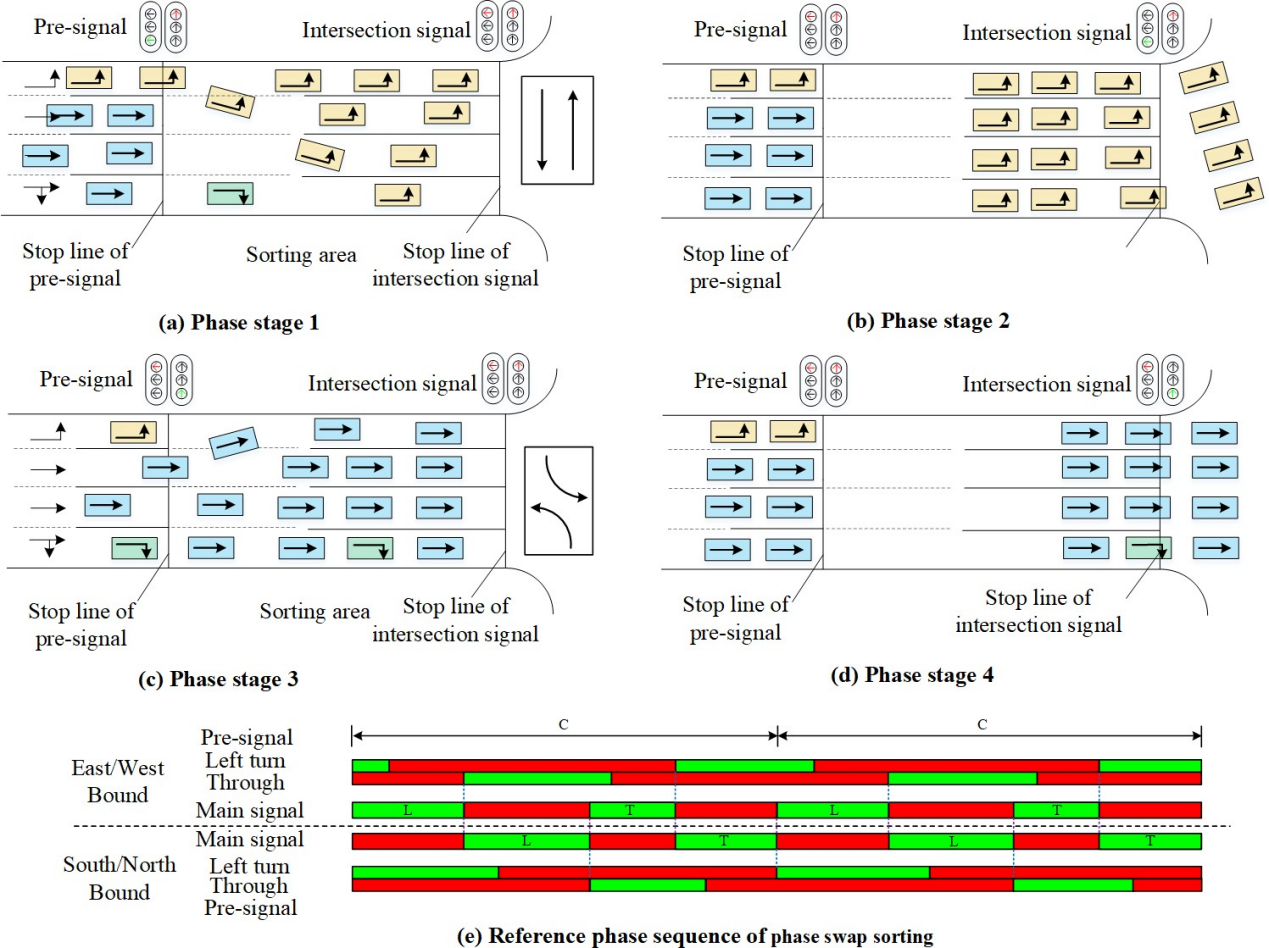
Components and operation of the pre-signal system with phase swap design.

The selection of the sorting strategy has a critical influence on the performance of the pre-signal system. Based on the sorting strategies for the sorting area, the signal timing methods have the tandem sorting [5, 6, 15] and the phase swap sorting[7, 16]. As shown in Fig 2, the tandem sorting will discharge the traffic in the sequence of eastbound/westbound LVs and TVs, and then switch to the southbound/northbound LVs and TVs. Both LVs and TVs of the same approach will be tandemly stored in the sorting area. The design of phase swap sorting strategy is shown in Fig 3. Under this sorting strategy, the main green phase will be “swapped” between the intersecting directions, which makes the traffic be discharged in the sequence of eastbound/westbound LVs, southbound/northbound LVs, eastbound/westbound TVs, and southbound/northbound TVs. The sorting area must be completely cleared at the end of each main green phase stage, so that the traffic to be serviced in the next main green phase stage can enter the sorting area during the main green phase stage for the crossing direction. The movement queued with fewer lanes before the pre-signal can have a longer time to enter the sorting zone under the tandem sorting design. However, the vehicles heading to different directions in the sorting area may block each other, which may reduce the overall efficiency. The tandem design also needs a longer sorting area to store the vehicles waiting for the main green, which will increase the delay. The vehicles can enter the sorting area using the green duration of the crossing direction under phase swap sorting strategy. The temporal and spatial utilization of the phase swap sorting strategy will be better than the tandem sorting.

Compared with other spatial traffic management measures, such as median U-turns, jug-handles, superstreets, continuous flow intersections and bowties[22–24], a pre-signal system is relatively easy to implement in the urban intersection. The geometric design of sorting areas, which includes lane allocation and length determination, is the primary work of designing pre-signal systems. Besides the Binary-Mixed-Integer-Linear-Programming (BMILP) model, the cellular automaton model[25] was also applied to optimize the length of the sorting area. The literature listed above recommends a length of 50–100 m for a sorting area with three lanes. The length of 50 m was calculated using expected traffic demand, but it will not be enough when the traffic demand exceeds this value. Limited method has considerations on the stochastic property of traffic demand, which may lead to inefficient utilization of the sorting area or insufficient sorting space. If the sorting area is too short, queue spillback will frequently occur when the arrival flow is heavy. At this time, the phase stages of the signal timing must be switched frequently, which also leads to higher lost time. On the other hand, if the sorting area is too long, the road space cannot be fully utilized, which will reduce the overall efficiency.

Little research has focused on the performance in terms of the spatial utilization of the road within the pre-signal system in consideration of the stochastic arrival property. These two gaps are summarized as follows.

1. Though the existing research can provide appropriate signal timing plans in multiple ways, the overall performance of the pre-signal system cannot be improved unless the geometric design is suitable. The existing research[5, 19] has determined the length of the sorting area using the largest number of vehicles that the main green phase stage can discharge. However, the furthest point that a queue can reach will be longer than the maximum queue length under static conditions. Because the furthest point of the queue will continually move further on the process of queue dispersion, the geometric design based on the maximum queue length can still experience queue spillback. Thus, the dynamic queue formation and dispersion process should be carefully modeled to determine an appropriate length of the sorting area for the purpose of improving operational efficiency and reducing the probability of queue spillbacks.
2. Most existing recommendations are determined by the expected value of the traffic demand, which may fail to service almost half the traffic demand in the stochastic arrival process. If the geometric design parameters are calculated using this expected value, there will be a high probability of queue spillback in both the upstream area and sorting area. As a result, a general guideline is urgently needed for determining the appropriate geometric design of the pre-signal system.

### Objective and contributions

The objective of this study is to develop a compound model for the geometric design of the pre-signal system to maximize its benefits. In consideration of various traffic demands and their stochastic properties, the traffic dynamics in both the upstream area and sorting area will be modeled using queue theory and shockwave theory, respectively. The minimum length of each component of the pre-signal system can be estimated by calculating the traffic status and boundary conditions, which are related to the traffic demand.

The major contributions of this study are twofold: (1) A series of analytical methods are established to describe the traffic dynamic in the pre-signal system based on the characteristics of each component. Queue theory is utilized to describe the queue generation process with Poisson arrivals behind the pre-signal stop line. The shockwave model meets the characteristics of traffic flow in the sorting area with many constraints. (2) The characteristics of the random arrival process and queue dispersion process are considered, which helps ensure that the proposed design of the pre-signal system will meet the requirements of traffic demand higher than a specific threshold.

The remainder of this paper is organized as follows. Section 2 analyzes the traffic dynamic of the upstream area, the keep-clear area and the sorting area of the pre-signal system. The method to obtain the geometric design of the pre-signal system is proposed in section 3. Section 4 evaluates the proposed geometric design plan through traffic simulation. Section 5 concludes the paper.

## Queuing process analysis of the pre-signal system

### Design considerations

There are two queues within each approach of the pre-signal system: the one behind the main stop line and the one behind the pre-signal stop line. We need to determine the geometric parameters related to the road space available to park those queues.

The sorting area is mostly utilized for LV and TV to allocate the right-of-way in the conflicting area. Since there is no conflicting point between the right turning vehicles (RV) and other motorized movements, the permitted control strategy is usually chosen for RV; that is, the right turning vehicle can enter the intersection on red when there is no conflict. However, the RVs should be queued in the sorting area with LVs or TVs when the pre-signal system is of the full utilization type. In case of a large right turning demand, an exclusive right turning lane with a partial utilization type signal is recommended.

To avoid deadlock in the conflicting area of the intersection and sorting area, all the vehicles in these areas must be cleared at the end of each phase stage, which means the number of exit lanes should be greater or equal to the number of lanes in the sorting area and the capacity of the sorting area should be large enough to park all the vehicles advanced into it. There should also be a relatively long inter-green interval for the phase swap strategy. The main green phase stage should be long enough to clear all the vehicles queued in the sorting area. The upstream area should also be long enough to prevent spillback to the upstream intersection. In this way, the following assumptions are considered in the proposed method.

- The right turning traffic demand is relatively low. All the RVs will be queued in the sorting area with LVs or TVs until they receive right-of-way. The traffic demand of RVs is added to the demands of LVs and TVs respectively. The proportion will be determined by the duration of pre-signal green for LVs and TVs. Otherwise, they should have an exclusive lane and have no influence on other vehicles.
- The capacity of the exit lanes should be greater than that of the sorting area to guarantee that all the vehicles leaving the sorting area can be discharged. The downstream intersection will not have spillback.
- The drivers will follow instructions. No deadlock happens in the pre-signal system.

### Traffic dynamic of the pre-signal system

**Fig 4:**
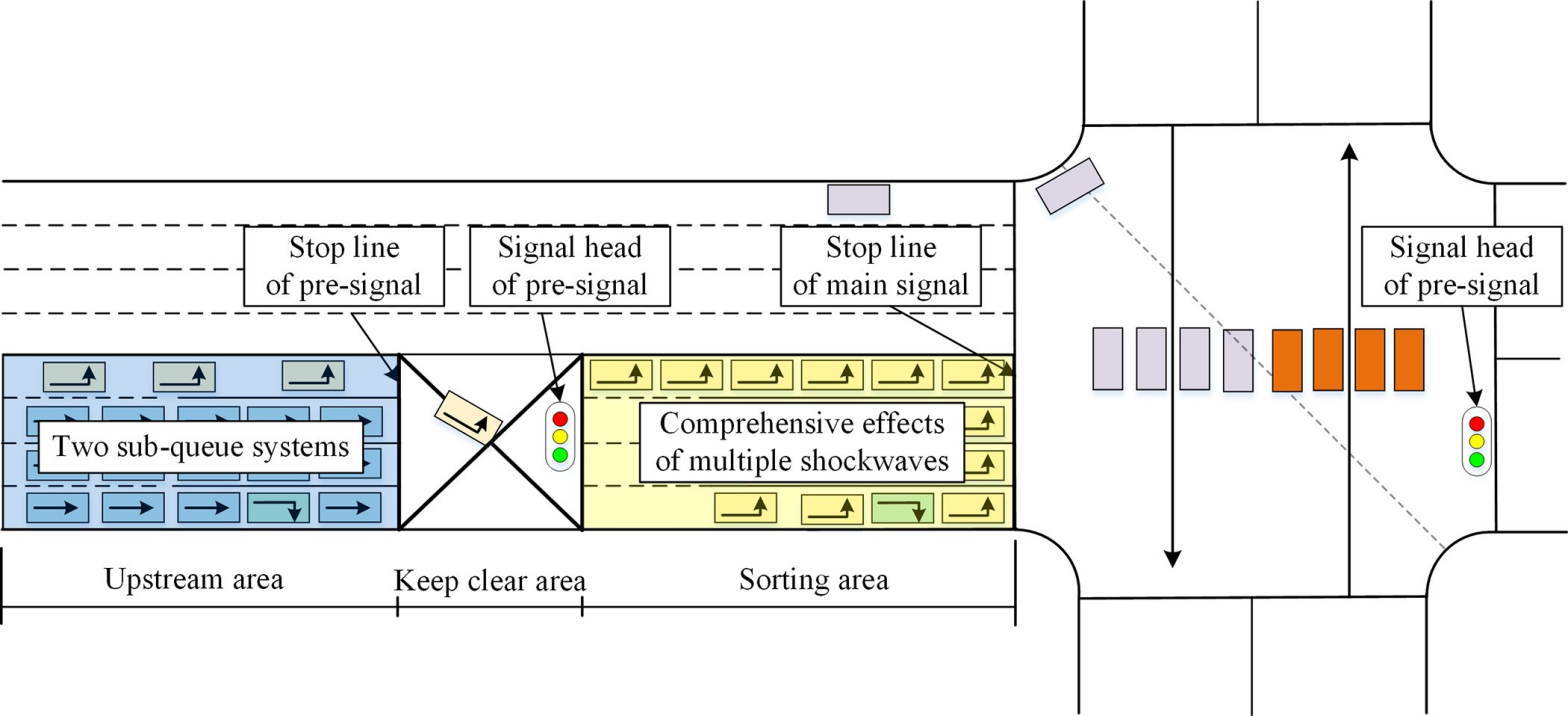
Layout and traffic dynamic analysis of the pre-signal system.

As illustrated in Fig 4, the pre-signal system comprises the sorting area and the upstream area. A keep-clear area should be set between the sorting area and the stop line of the pre-signal to provide enough space for drivers to read the signal indications and finish the lane changing process. Based on these criteria for setting the traffic signal devices, the minimum sight distance for signal visibility should be no less than 50 meters[26]. The length of the keep-clear area should be longer than this criterion; otherwise, a warning sign must be installed. The upstream area should provide enough space for the queued vehicles to finish their corresponding lane changing processes. In this way, the minimum length of the upstream area can be estimated by calculating the queue length and the distances needed for lane changing behaviors. The sorting area is used to store the transient queues formed by the different offsets and phase stages of the pre-signal and main signal. The length of the sorting area and its lane allocations decide the storage capacity of the sorting area. Every effort should be made to ensure these transient queues do not spill back to the pre-signal and can be totally discharged during the next green phase stage of the main signal. The length of the sorting area and related lane allocation areas is another major geometric design task for the pre-signal system.

The characteristics of the arriving flow at the upstream area are affected by the traffic status and signal timing plan of the upstream intersection, as well as the geometric design of the road segment. The platoon discharged from the upstream intersection will become dispersive, and have stochastic property when they arrive the pre-signal system. In this way, queue theory is an appropriate model to model the queue process behind the pre-signal stop lines, which can be modeled by two sub-queuing systems. The longer queue length for LVs and TVs can be utilized to determine the appropriate length of the upstream area. The queue within the sorting area is highly controlled by the signal coordination plan and geometric design. Thus, the traffic in the sorting area has little stochastic property, which makes shockwave an ideal way to model it. There will be multiple shockwaves generated during the queuing process. The maximum queue length can then be determined by considering the boundary conditions of these shockwaves.

In the queuing system, the stop line is the server. Because of the no-memory property of the exponential distribution[27], the Poisson model is the most commonly used model to describe the uncontrolled arrival process[19]. For the reason that the arriving traffic must finish the lane changing and queuing process within the upstream area, we should set enough road space for the upstream area. Thus, the arrival flows in the upstream road segment is considered as Poisson flows with arrival rates of *λ*_*l*_ and *λ*_*t*_ for LVs and TVs (with RVs), respectively. The queued vehicles will travel through the stop line during the green phase stage. We assume that the service time during the green phase stage follows an exponential distribution with service rate *μ*, which can be calculated by Eq (1). If there are lanes for one specific movement before the stop line, the *k* vehicles will cross the stop line almost at the same time. In other words, the vehicles will pass the stop line in batches. In the partial-batch model, the server can process partial batches up to a maximum size of *k*. The service time for each batch follows an exponential distribution. This type of service is termed bulk service. This queuing system can be described using the notation M/M^[k]^/1. The bulk service queue model can be utilized to describe the queue before the pre-signal. We have

$$\mu =s\cdot \frac{g}{C}$$ (1)

where *μ* is the service rate for one lane (*pcu*/*h*), *s* is the saturation flow rate (*pcu*/*h*), *g* is the corresponding green duration (*s*), and *C* is the cycle length (*s*).

Because of the filtering process of the pre-signal, there will be several shockwaves generated during the formation and dissipation process of the queue. These shockwaves are highly related to the signal timing plan, geometric design and traffic dynamics within the sorting area. Most traffic states within the pre-signal system are stable, which makes shockwave theory an ideal way to estimate the queuing process with the given signal timing and geometric design. The furthest point that a queue can reach can then be calculated by considering the boundary conditions. The optimal length of the sorting area can then be estimated by considering the variations in traffic demand.

## Geometric design method for the pre-signal system

### Framework

**Fig 5:**
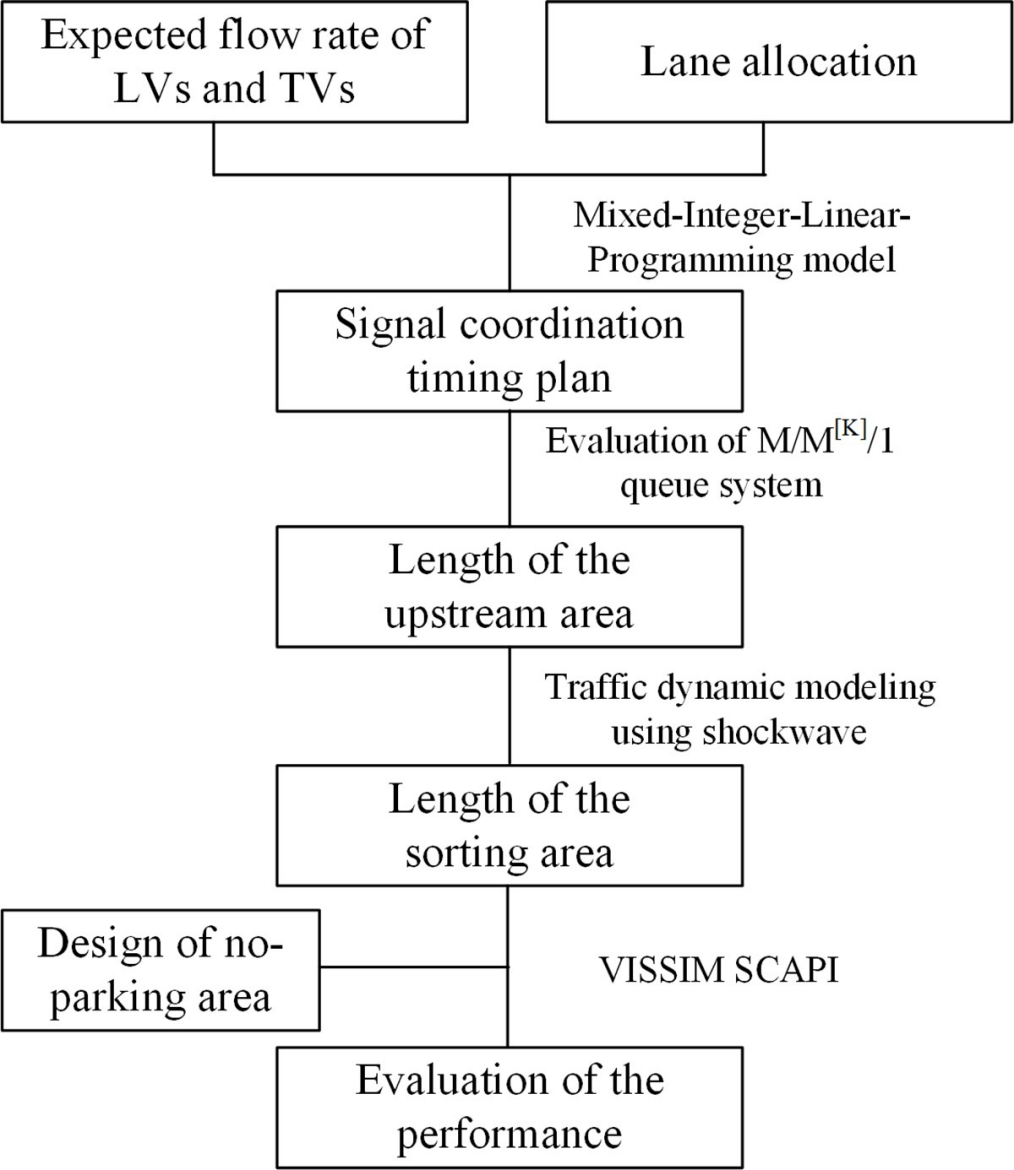
Framework of the geometric design method.

The performance of the pre-signal system is highly related to the corresponding signal timing plan. A reference timing plan must be provided to process the geometric design method. If the fixed timing method is selected, the signal timing can be optimized using the Mixed-Integer-Linear-Programming method proposed by Yan[7]. When the dynamic timing method is applied, we can use the signal timing plan from Yan’s method as the maximum likelihood reference timing plan. With the reference timing plan, the required lengths for the upstream area and the sorting area can be calculated using queue theory and shockwave theory, respectively. Considering the stochastic property of arriving flow, the geometric design parameters should meet traffic demand that is higher than a specific percentage. The effectiveness of the geometric design can be evaluated using the VISSIM with Signal Control Application Programming Interfaces (SCAPI)^[^^28^^]^. If the efficiency of the pre-signal system can meet the requirements under the given traffic demand, the geometric design plan can be accepted. Otherwise, the proposed plan needs to be adjusted. The whole process is summarized in Fig 5.

### Length of the upstream area

The upstream road segment with the pre-signal is composed of two independent queueing systems: one for LVs and the other one for TVs. The upstream road segment should be longer than the sums of the lengths of the queue generated by the pre-signal and the additional road space for lane changing behaviors and decelerations. The average queue length during the green phase stage can be directly estimated by the queuing system, and the queue formed during the red indication can be calculated according to the arrival process. The worst condition between these two sub-queuing systems will be selected to determine the minimum length of the upstream area.

The queuing system at the pre-signal during the green phase stage is an M/M^[k]^/1 queuing system, which is essentially equivalent to the Erlang arrival queue; that is, the M/M^[k]^/1 queuing system is equivalent to the E_k_/M/1 queuing system. We can take advantage of these two types of queuing systems to determine the queue length for a specific condition.

If the arrival rate for an intersection approach with *k* lanes is *kλ* and the interarrival times are exponentially distributed, the Poisson distribution can be utilized to describe the arrival flow. Since the distributaries of a Poisson flow and the confluence of several Poisson flows are also Poisson distributed, we can consider at *k* arrivals with exponentially distributed interarrival times. Then, the interarrival times are Erlang type-k distributed with a mean of 1/*λ*. As this system is identical in structure to the full-batch bulk-service model, we can obtain the probabilities *p*_n_, denoting the number of customers in the system in a steady state, by Eq (2):

$$p_{n}=\sum_{j=nk}^{nk+k-1}p_{j}^{\left(P\right)}=\rho (1-r_{0}^{k}){\left(r_{0}^{k}\right)}^{n-1}$$ (2)

Here, *ρ* is the traffic intensity and *r*_0_ is the single root in (0, 1) of the characteristic equation shown by Eq (3):

$$\mu r^{k+1}-(k\lambda +\mu )r+k\lambda =0$$ (3)

Like the M/M/1 queue, the Eq (2) has a geometric form, with $r_{0}^{k}$ as the geometric parameter instead of *ρ*. Based on the characteristics of the queue with general-time steady-state system-size probabilities, the queue length of the E_k_/M/1 queue can be calculated by Eq (4):

$$L=\rho (1-r_{0}^{k})\sum_{n=1}^{\infty }n{\left(r_{0}^{k}\right)}^{n-1}=\rho (1-r_{0}^{k})\frac{1}{{\left(1-r_{0}^{k}\right)}^{2}}=\frac{\rho }{1-r_{0}^{k}}$$ (4)

The queue generated during the red phase stage equals the number of vehicles arriving at the stop line during the red phase stage, which can be calculated using the characteristics of the Poisson flow. Therefore, the probability of *N*_*r*_ arrivals occurring during the red duration *r* can be calculated using Eq (5), and the cumulative probability is shown in Eq (6). If a queue length under a specific percentage is needed, we can calculate the number of arrivals when the cumulative probability is less than the selected threshold:

$$P(N_{r}=n)=\frac{e^{-k\lambda r}{\left(k\lambda r\right)}^{n}}{n!}$$ (5)

$$C(N_{r}=n)=\sum_{i=1}^{n}P(N_{r}=i)$$ (6)

In conclusion, the minimum length of the upstream area of the pre-signal system can be determined by summing the average queue lengths during the green/red phase stage and the road spaces needed for lane changing and decelerating, which have been well studied[29].

### Design of the sorting area

The traffic dynamic in the sorting area is highly related to the geometric design, the signal timing of the main signal and the pre-signal. Suppose the vehicles entering the sorting area are from *k* lanes at the pre-signal and there are *j* lanes in the sorting area (*j*>*k*). Thus, when the queuing system at the pre-signal is stable (*λC*≤*μg*_*p*_), the expected number of vehicles that can enter the sorting area should equal the number of arrivals of the queuing system. If the number of arrivals is greater than the maximum number of departures in the pre-signal green phase stage (*λC*>*μg*_*p*_), the model will turn to be the production of the saturation flow rate, the number of lanes and the duration of the pre-signal green phase stage (see Eq (7)):

$$N_{s}=\{\begin{array}{cc} k\lambda C & \lambda C\leq \mu g_{p}\\ k\mu g_{p}=ks\frac{g_{p}^{2}}{C} & \lambda C>\mu g_{p} \end{array}$$ (7)

**Fig 6:**
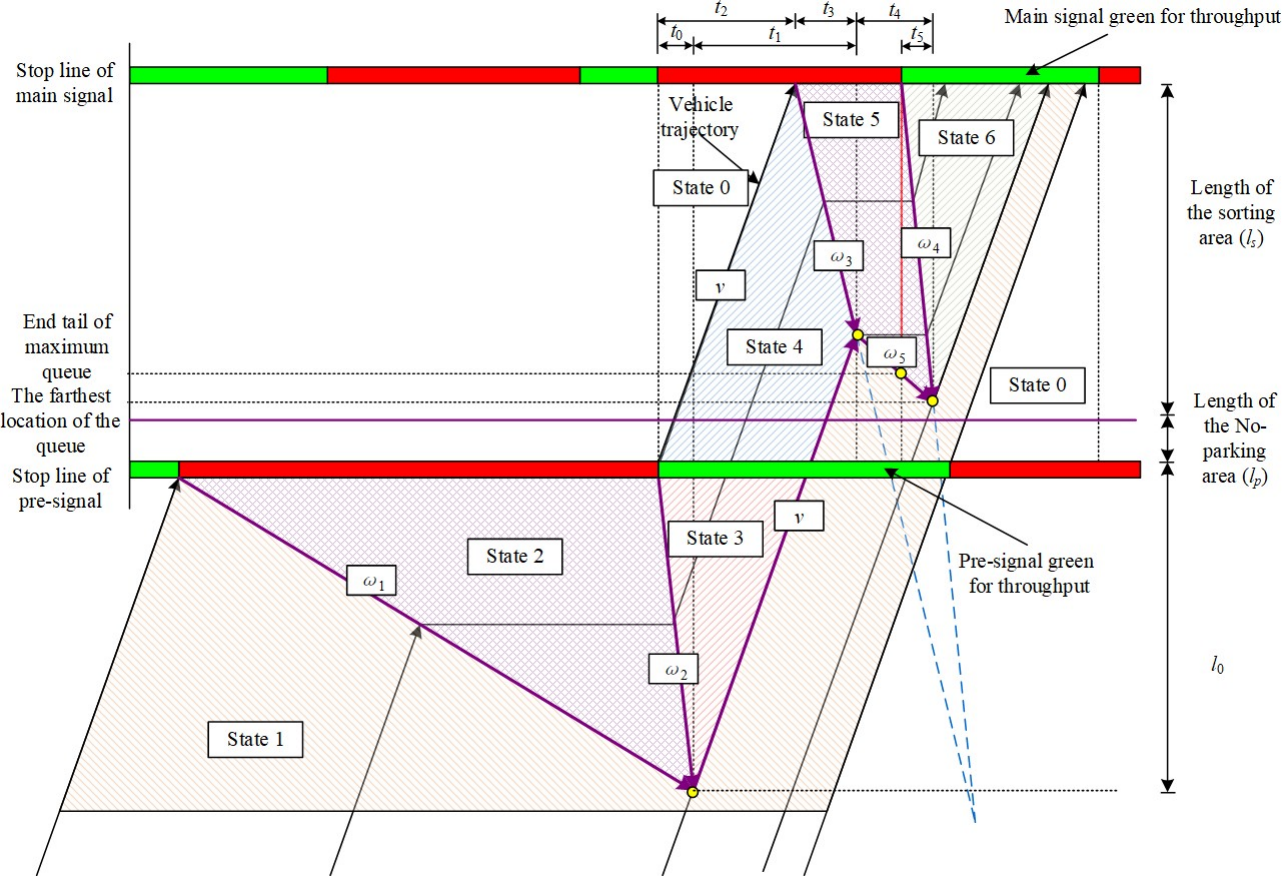
Time-space diagram of the pre-signal system (Type 1).

The time-space continuum of the traffic dynamic within the sorting area can be analyzed using shockwave theory. Based on the discharge flow rate of the pre-signal, the time-space continuum of the traffic dynamic within the sorting area can be divided into two categories. When the queuing system at the pre-signal is stable, the service rate at the beginning of the green phase stage will be the saturation flow rate *s*. When the queue is cleared, the vehicles will be discharged at the arrive rate *kλ*. Fig 6 illustrates the time-space diagram for the throughput movement within one cycle. There are six traffic states with six related shockwaves. When the pre-signal turns red, the vehicles will begin to form a queue in the upstream area. At this time, a shockwave with speed *ω*_1_ will be generated to separate the arrival flow (state 1) and the queue (state 2). The queue will be discharged at the saturation flow rate at the beginning of the green phase stage of the pre-signal (state 3: there are *k* lanes behind the pre-signal stop line). When the vehicles cross the stop line at the pre-signal, the number of lanes increases to *j* from *k*. The density is then reduced to *k*/*j* of the density in state 3 with the same flow rate (state 4). The main signal will be red when the leading vehicles arrive at its stop line. The queue will be formed again within the sorting area (state 5). At this time, a backward shockwave with speed *ω*_3_ will be generated and will meet the forward shockwave generated at the point where the queue in the upstream area is fully cleared. When these two shockwaves meet, another backward shockwave with speed *ω*_5_ (*ω*_3_>*ω*_5_ = *ω*_1_) will be generated. The pre-signal green phase stage will end early. At this time, the speed of shockwave *ω*_5_ will be zero (details will be discussed in section 4.3). When this shockwave meets the backward shockwave generated at the beginning of the main green phase stage, the queue within the sorting area will be completely cleared. The queue within the sorting area will be discharged with a flow rate of *js* and the optimum density (state 6). The shockwave between state 5 and state 6 will propagate backward at speed *ω*_4_. The flow rate and density of each state are listed in Table 1. Since all the vehicles in the pre-signal system are traveling with limitations, we can assume that these vehicles travel at a constant speed *v*.

**Table 1:** Parameters for each traffic state within the pre-signal system.

Traffic state	State 1	State 2	State 3	State 4	State 5	State 6
Flow rate	*K*·*λ*	0	*k*·*s*	*k*·*s*	0	*j*·*s*
Density	*k*·*λ*/*v*	*k*_*j*_	*k*_*m*_	*k*/*j k*_*m*_	*k*_*j*_	*k*_*m*_

*k* and *j* are the numbers of lanes of the upstream area and the sorting area, respectively. *k*_*j*_ is the jam density and *k*_*m*_ is the optimum density, which is the density when the flow rate reaches its capacity.

Eqs (8) to (18) can be established to describe the time-space relationships of the traffic dynamics within a type 1 pre-signal system. The modeling of traffic dynamics should be divided into three parts. Eqs (9) to (11) describe the relationships between state 1, state 2 and state 3. Eqs (12) to (14) describe the relationship between state 3 and state 4. Eqs (15) and (16) describe the relationship between state 5 and state 6. As shown in Eq (17), we can then acquire the relationship between the length of the sorting area (*l*_*s*_) and the furthest point that the queue can reach (*l*_*q*_). Eq (17) indicates that the longer the sorting area, the shorter the maximum queue length. Since the length of the sorting area should be greater or equal to the maximum length of the queue, we can then calculate the extreme length of the sorting area by assuming these two lengths are the same, as shown in Eq (18).

$$\omega =\frac{\Delta q}{\Delta k}$$ (8)

$$r_{p}+\frac{l_{0}}{\omega_{2}}=-\frac{l_{0}}{\omega_{1}}$$ (9)

$$l_{0}=-\frac{\omega_{1}\omega_{2}r_{p}}{\omega_{2}+\omega_{1}}$$ (10)

$$t_{0}=-\frac{\omega_{1}r_{p}}{\omega_{2}+\omega_{1}}$$ (11)

$$t_{2}=\frac{l_{s}+l_{p}}{v}$$ (12)

$$t_{0}+t_{1}=t_{2}+t_{3}$$ (13)

$$vt_{1}-\omega_{3}t_{3}=l_{p}+l_{s}+l_{0}$$ (14)

$$t_{2}+t_{3}+t_{4}-t_{5}=r_{m}$$ (15)

$$\omega_{3}t_{3}+\omega_{1}t_{4}=\omega_{4}t_{5}$$ (16)

$$l=\omega_{4}t_{5}=\frac{\omega_{1}\omega_{4}(\omega_{1}-\omega_{3})(\omega_{1}+v)}{\left(\omega_{4}+\omega_{1})(\omega_{2}+\omega_{1})(v-\omega_{3}\right)}\cdot r_{p}-\frac{\omega_{1}\omega_{4}}{\left(\omega_{4}+\omega_{1}\right)}\cdot r_{m}+\frac{\omega_{1}\omega_{4}}{\omega_{4}+\omega_{1}}\cdot \frac{l_{s}+l_{p}}{v}$$ (17)

$$l=\frac{\omega_{1}\omega_{4}}{v(\omega_{1}+\omega_{4})-\omega_{1}\omega_{4}}\left[\frac{v(\omega_{1}-\omega_{3})(\omega_{1}+v)}{\left(\omega_{1}+\omega_{2})(v-\omega_{3}\right)}\cdot r_{p}-\frac{v}{\omega_{1}+\omega_{4}}\cdot r_{m}+\frac{l_{p}}{\omega_{1}+\omega_{4}}\right]$$ (18)

**Fig 7:**
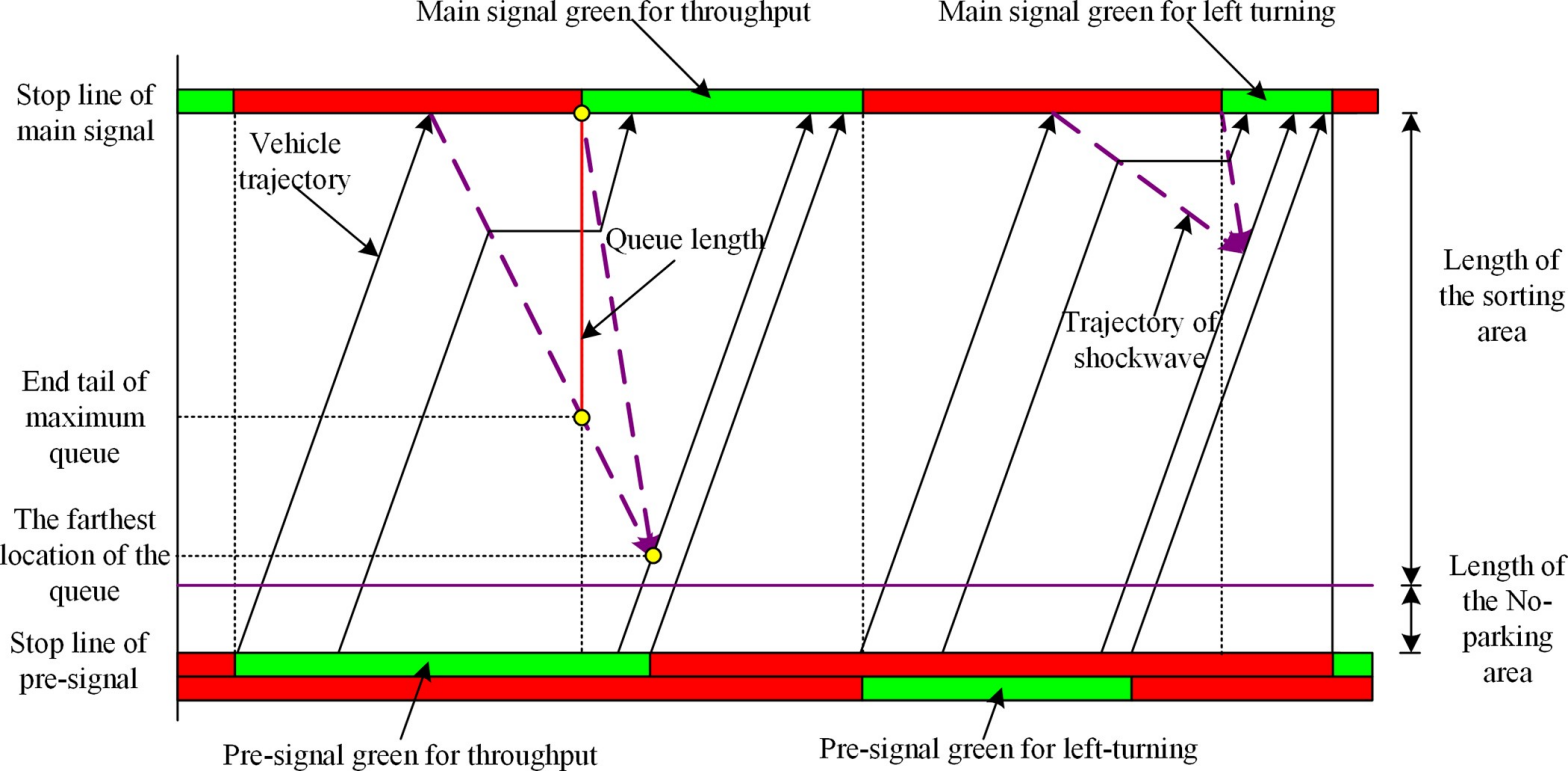
Time-space diagram of the pre-signal system (Type 2).

If the pre-signal discharges flow at the saturation flow rate during all the green phase stages, the time-space continuum of the traffic dynamic within the sorting area can be shown as Fig 7. The sorting area should be long enough to store all the vehicles discharged by the pre-signal. In other words, the two shockwaves (*ω*_3_ and *ω*_4_) must meet within the sorting area (see Fig 7). In a similar way to the modeling process described previously, we can obtain the relationship shown in Eq (19). The minimum length of the sorting area can then be calculated by Eq (20).

$$r_{m}-\frac{l_{s}+l_{p}}{v}=-L\left(\frac{\frac{k}{j}k_{m}-k_{j}}{q_{m}-0}-\frac{k_{m}-k_{j}}{q_{m}^{\prime }-0}\right)=\frac{(j-k)k_{j}}{kjs}L$$ (19)

$$l=\left(r_{m}-\frac{l_{p}}{v}\right)\frac{j\cdot k\cdot s\cdot v}{(j-k)k_{j}v+jks}$$ (20)

## Case studies

### Experimental design

**Fig 8:**
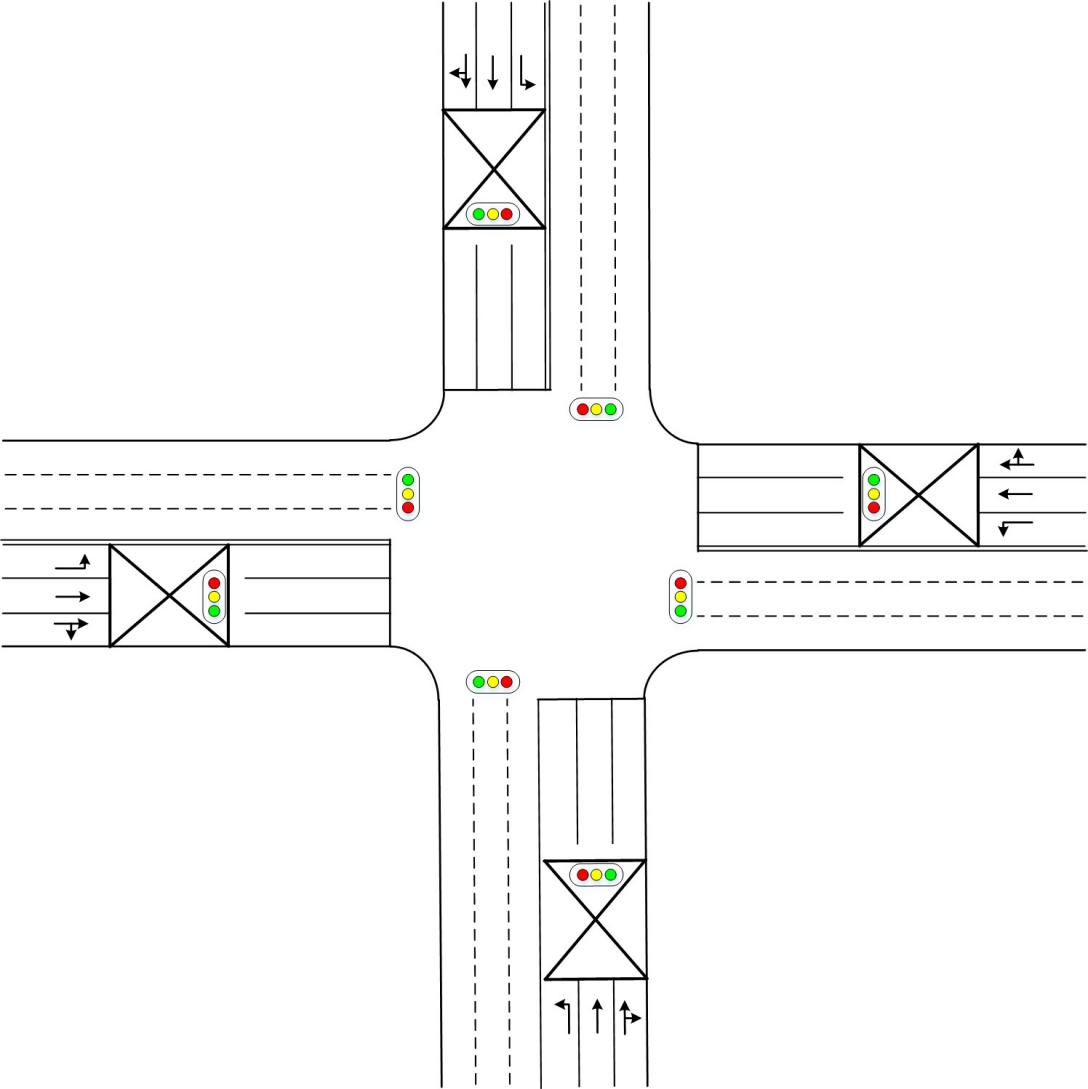
Layout of the tested intersection.

As shown in Fig 8, a four-arm intersection with a pre-signal installed at each approach is selected to optimize the geometric design. There are three lanes at each approach. Two lanes before the stop line of the pre-signal are utilized for the TVs and RVs, while the remaining lane services the LVs. Assuming there are no special vehicles, the full utilization type sorting area is selected. As shown in Table 2, three scenarios with corresponding expected traffic demands are selected to test the proposed method. Scenario 1 is the congested traffic state, Scenario 3 is the free-flow traffic state, and somewhere in between is the traffic state of Scenario 2. The arrivals of the LVs and TVs (Assume the right turning demand is relatively small, and the demand of RVs is added to the demand of LVs and TVs according to the pre-signal green for each movement respectively) are assumed to follow Poisson distributions. Only the green and red phase stages (amber is counted as part of the green phase stage) are considered for decision making. Since each intersection approach has three lanes, a 25-meter length is selected for the keep-clear area. The average vehicle speed in the sorting area is set to 8 m/s and the saturation flow rate is set to 1800 pc/(h·ln).

**Table 2:** Traffic demand in the three test scenarios (unit: pcu/h).

Movement		Scenario 1	Scenario 2	Scenario 3
Eastbound	Left	600	400	200
	Throughput	1200	800	400
Westbound	Left	600	400	200
	Throughput	1200	800	400
Southbound	Left	600	400	200
	Throughput	1200	800	400
Northbound	Left	600	400	200
	Throughput	1200	800	400

**Fig 9:**
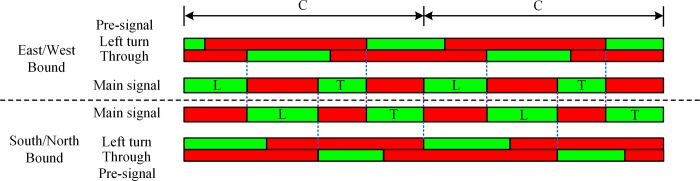
Reference phase sequence of the pre-signal system.

The signal timing of the pre-signal system was optimized using the Mixed-Integer-Linear-Programming method proposed by Yan[7]. The proposed reference phase sequence is shown in Fig 9 and the duration of each phase stage (green phase stage and previous red phase stage) is listed in Table 3. We modified the phase sequence from the original phase plan shown in Fig 9 in Yan et al[7] by converting the amber phase stages to green phase stages and combining phase stages indicating the same signal head; the signal phase plan shown in Fig 9 is consistent with those in Yan’s paper[7]. We use the modified phase sequences to evaluate the performance of each geometric design plan.

**Table 3:** Reference signal timing plans (unit: *s*).

Signal phase stages (duration of green and previous red)	Scenario 1	Scenario 2	Scenario 3
Main signal east/west left	22/40	16/34	13/26
Main signal south/north left	34/22	35/16	25/13
Main signal east/west through	40/34	34/35	26/25
Main signal south/north through	40/40	34/34	26/26
Pre-signal east/west left	47/89	35/84	24/66
Pre-signal south/north left	41/90	36/83	23/67
Pre-signal east/west through	59/77	54/65	36/54
Pre-signal south/north through	65/71	53/66	37/53
Cycle length	136	119	90

**Fig 10:**
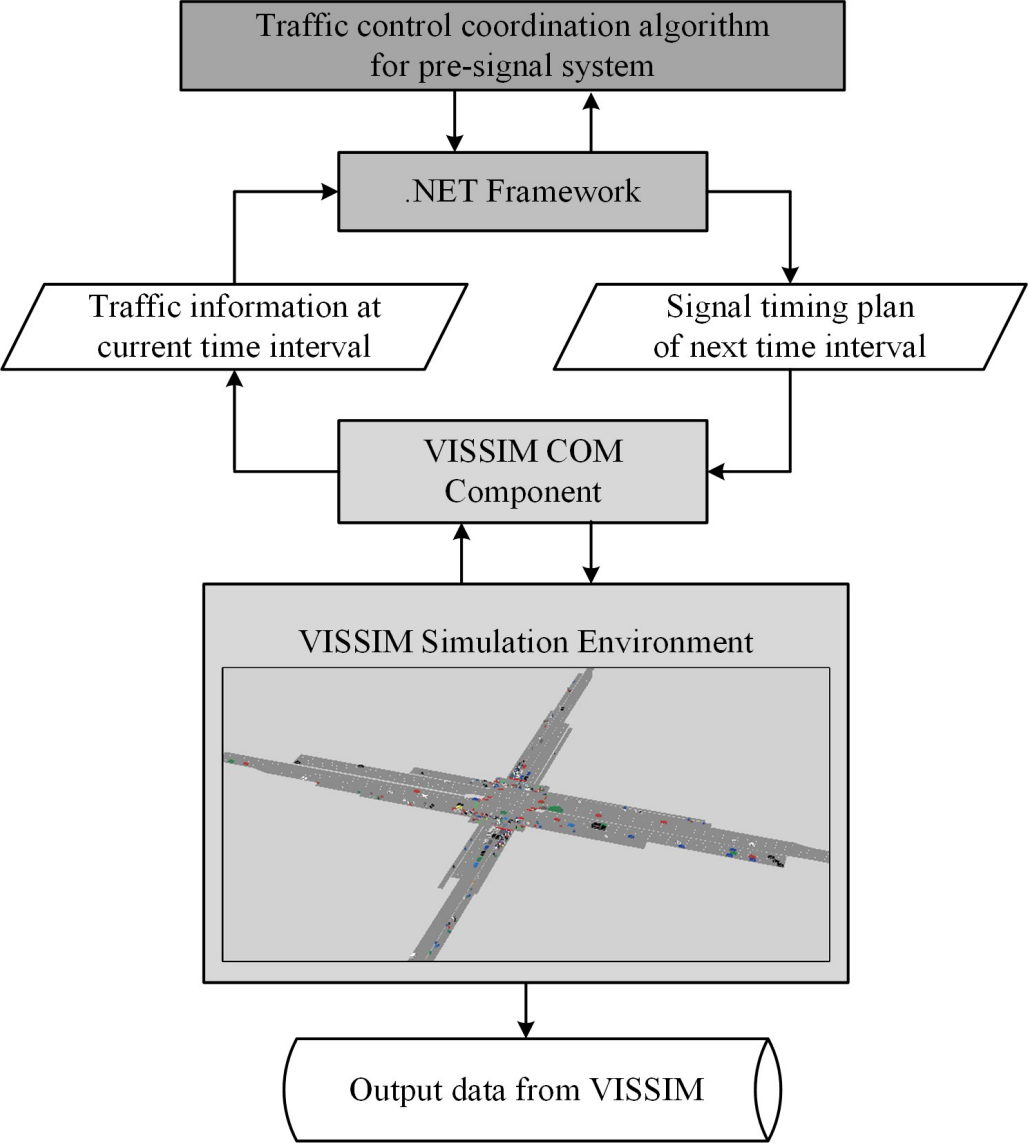
Framework of the VISSIM SCAPI environment.

The proposed designs under the scenarios listed above are evaluated using VISSIM Signal Control Application Programming Interfaces (SCAPI)[28]. The SCAPI were written in the C++/CLR language, and their framework is shown in Fig 10. The signal coordination algorithms for the pre-signal and intersection signal are embedded into a single dynamic link library (DLL) file. Real-time information, such as the counts and occupancies of the detectors, can be collected from VISSIM via the COM component. The information is then sent to the signal coordination algorithms via a .NET framework for optimizing signal timing information, such as the cycle length, split and offset to the next interval. The new desired phase tells the VISSIM how to control all the signal heads installed in the road network. Before the experiment, we used seven days high-resolution video data (Apr. 14, 2013—Apr. 21, 2013) from the northbound approach of the Xiaozhai intersection in Xi’an to calibrate the parameters in VISSIM. The SIMI Motion software was utilized to obtain the speed, acceleration, headway and lateral gap of selected vehicles. The vehicle class, desired speed, speed distribution, driving behaviors such as the car following model and the lane changing model in VISSIM were carefully adjusted to fit these patterns.

### Results

**Fig 11:**
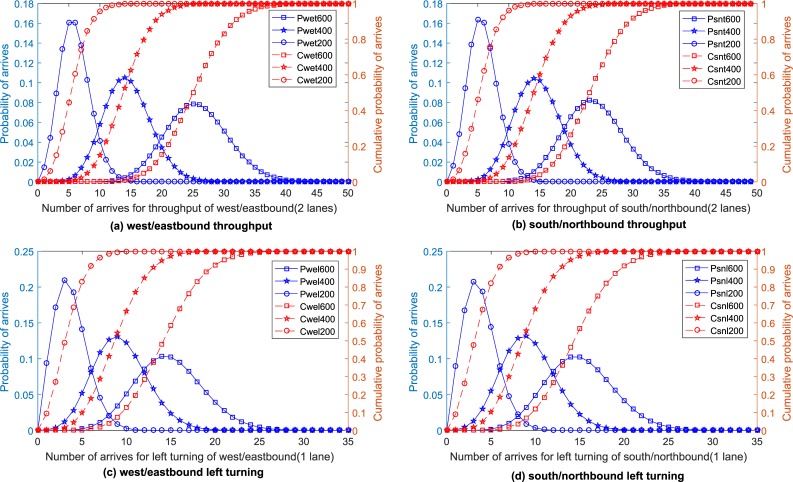
Probability and cumulative probability of selected arrivals.

Because the interarrival times at the upstream area are Erlang type-k distributed with mean 1/*λ* (see Table 2), the expected queue length during the pre-signal green and red phase stages can be calculated using Eqs (6) and (8), respectively. The queue lengths with cumulative probabilities of 85%, 90%, and 95% are selected to determine the necessary length of the upstream area; that is, there is a maximum probability of 15%, 10%, and 5%, respectively, that the queue will be longer than the length selected. The distributions of the queue for each movement are shown in Fig 11. The estimation results for the upstream queue are presented in Table 4.

**Table 4:** Determination of the minimum length of the pre-signal system.

Components	Movement	Scenario 1	Scenario 2	Scenario 3
Upstream area Green phase stage (pcu)	east/west left	1	0	0
	south/north left	1	0	0
	east/west through	1	1	0
	south/north through	1	1	0
Upstream area Red phase stage (pcu) (85%/90%/95%)	east/west left	21/20/19	15/13/12	7/6/6
	south/north left	22/20/19	14/13/12	8/6/6
	east/west through	17/16/15	10/10/9	5/5/4
	south/north through	16/15/14	10/10/9	5/5/4
Keep-clear area (m)	all	25	25	25
Sorting area (m) (Type 1 Expectation/ Type 1 85%/Type 2)	east/west left	63.9/70.0/115.8	50.6/58.9/97.2	30.1/39.3/72.5
	south/north left	43.7/45.6/60.2	36.8/41.5/41.7	23.0/29.5/32.4
	east/west through	81.0/85.8/180.3	68.1/76.6/186.0	42.8/54.7/128.8
	south/north through	93.4/100.7/214.6	76.8/87.5/180.3	47.4/61.0/134.5

Based on the analysis of the traffic dynamics in the sorting area, if the pre-signal can discharge vehicles at the saturated flow rate for the entire pre-signal green phase stage, the furthest points that the queue can reach are shown in Table 4 (Type 2). On the other hand, with the maximum main signal green phase stage and saturation flow rate, 20 vehicles can depart from one lane of the sorting area. Assuming the space headway under the queuing condition is 7 m, the length of the sorting area should be less than 140 m, which is smaller than the maximum length in Table 4. In this way, it can be seen that the pre-signal did not discharge the flow at the saturation flow rate for the entire green phase stage. Eq (18) should be chosen to locate the furthest point that the queue (type 1) can reach. This equation indicates that the minimum length of the sorting area is related to the arrival process, which is a random process. The minimum length of the sorting area can be calculated using the expected number of arrivals or a specific cumulative probability for arrivals. Table 4 shows the results for the minimum length of type 1 sorting area using the expectation for 85% of the arrivals. The minimum required length of the pre-signal system can be estimated by summing the minimum lengths of all the components. Once the geometric design of the pre-signal system is selected, it is difficult to change. Thus, the geometric design should meet the requirements of most conditions. In this way, relatively worse conditions (such as the 85% arrival condition) for each movement under all the scenarios should be selected to calculate the geometric design parameters of the corresponding intersection approaches. The minimum lengths of the upstream area and the sorting area of the tested intersection under 85% of the arrivals are 85.8 m for east/westbound and 100.7 m for south/northbound.

The VISSIM SCAPI was utilized to obtain the Measures of Effectiveness (MOE) for the proposed geometric design plan. Two geometric design plans were selected for comparison to the proposed plan (Plan 1). Plan 2 used the length determined by the maximum number of vehicles that can be discharged during the main green phase stage. Plan 3 is the conventional design obtained from Yan’s method[7] (the longest design of all the four approaches is selected. Assume the upstream area is long enough.). All three geometric design plans were tested using reference timing plans calculated through the MILP method for all three traffic demand scenarios shown in Table 2. For each scenario, we ran 20 simulations, each of length 3600 s. The average values of all the simulations for each scenario were recorded as the MOE. The occupancy rate of the detector at the main signal stop line (OCC) was selected as the performance indicator for the spatial utilization of the sorting area. The maximum queue length of the sorting area (MLS) and the maximum queue length behind the pre-signal stop line (MLP) were chosen as the performance measures for the queue formation process. The operational efficiency indicator used the commonly utilized average delay (AVD). The ratio of flow to saturation flow (RFS) was selected as an indicator that represents how the increased saturation flow is used with the current geometric design plan. All MOE indicators obtained by the simulation are listed in Table 5.

**Table 5:** Recommended design parameters and related MOE.

Design plans ID		Plan 1			Plan 2			Plan 3		
Design	*l*_*s*_(m)	105			140			85		
	*l*_*p*_(m)	25			25			25		
	*l*_*u*_(m)	>350			>350			>500		
MOE	Demand scenarios	1	2	3	1	2	3	1	2	3
	OCC(%)	68.6	36.1	13.2	61.1	32.6	12.8	64.2	39.8	20.6
	AVD(s)	100.8	77.5	42.1	103.6	78.3	41.5	187.6	115.8	51.4
	MQP(m)	268	150	62	271	147	64	399	238	105
	MQS(m)	102	86	39	101	85	39	85	85	41
	RFS	0.832	0.631	0.396	0.781	0.615	0.390	0.612	0.593	0.423

Although the reference signal timing plans were calculated based the geometric design of plan 3, which did not consider the queue dispersion process within the sorting area, almost all the queues in the simulation with demand 1 and 2 spill out. The simulation results of plan 3 indicate that the conventional design may get a short sorting area. Although the OCC was high in plan 3, the AVD, MQP, and MQS were also higher than in the other plans, which means the whole system was in a congested state with lower spatial utilization and efficiency. The number of throughput vehicles was much lower than the demand, it led to a relatively low RFS, which means the increased capacity given by the pre-signal was not effectively used. The results of the simulations indicate that the signal timing plan must be coordinated with the geometric design. Otherwise, the overall efficiency will be significantly diminished.

The efficiency indicators for plan 1 is similar to those of plan 2. However, the spatial utilization rate of plan 2 is lower with a longer delay, which means the proposed geometric design can improve spatial utilization without reducing the overall efficiency.

### Discussion

Based on the evaluation of the queue at the upstream area, the queue generated during the pre-signal green phase stage is much shorter than the one generated during the pre-signal red phase stage. When the pre-signal is a stable system (*λC*≤*μg*_*p*_), the queue should be cleared at the end of each green phase stage. However, when the number of arrivals exceeds the capacity, the pre-signal faces an over-saturation condition. At this time, there will be a residual queue after each cycle. This queue will continue to grow until the occurrence of a detrimental effect such as “spillback” or “deadlock”. Compared to the arrivals during the red phase stage, the expectation of arrivals at an arrival rate of 600 pcu/(h·ln) will be approximately 3 vehicles smaller than for the queue with a cumulative probability of 85%. In this way, if the length of the upstream area is determined using the expected value, there will be a probability of 15% that some additional storage road space of more than 21 meters is needed. In the modeling process, we also assumed that the vehicles at the pre-signal were in the appropriate lanes. Thus, an additional lane changing distance should be included in the upstream area; it usually should be at least 200 meters for a speed limit of 60 km/h. Based on the estimations of the queue length behind the pre-signal stop line, the upstream area of the studied intersection is suggested to be no less than 350 meters.

The keep-clear area should provide enough space for the drivers to read the signals and finish the lane changing process to the sorting area. The keep-clear area can be considered an invalid area between the two stop lines; it should be minimized as much as possible. Twenty meters is the minimum length, but it should be longer when the intersection has more approach lanes.

When we discussed the queue status in the sorting area, we proposed two types of traffic conditions: the queue behind the pre-signal is long enough to discharge at the saturation flow rate (type 2) or it is not (type 1). In our experiments, the speed of the backward shockwave generated by the arriving vehicles approximately equals the approach speed. If the previous main red phase stage is longer than twice the approach time (*t*_2_ in Fig 5), the furthest point in the queue will pass the pre-signal stop line. At this time, the pre-signal green phase stage must be terminated early to meter the traffic entering the sorting area, which will turn the traffic condition into type 1 (the speed of the shockwave generated at the furthest point will be zero). Based on Eq (9), the highest arrival rate for type 1 condition is *μg*_*p*_/*C*. The lengths of the sorting areas obtained by Eq (20) and (22) are the minimum lengths under the corresponding traffic condition. Traffic engineers in the field should consider adding additional length.

According to the MOE from the simulation, the pre-signal system has the best performance under relatively congested conditions. The occupancy rates under the demand scenarios 2 and 3 are smaller than under scenario 1, which means the utilization of the sorting area decreases as the traffic demand decreases. At this time, the dynamic signal timing method will be better for the medium traffic demand condition. The pre-signal can be temporarily disabled to reduce the delay and the stop time when the traffic demand is low. Considering the constraints listed above, the length of the sorting area and the upstream area in our experiment could be set to 110 m and no less than 350 m respectively.

In real-world applications, a reference signal timing plan and geometric design can be obtained using BMILP model with observed traffic demand. The furthest point that the queue can be calculated using the proposed model. The length of the sorting area can then be determined according to the affected area of the queue in the sorting area. The length of the upstream area should be no less than the summation of queue affected area and necessary space for lane changing and decelerating. The keep clear area is suggested to be designed based on the lane number of lanes before the pre-signal stop line. The warning signs should be installed if necessary.

## Conclusions

This paper focuses on the development of a geometric design method for an intersection with tandemly installed pre-signals. The queue theory and shockwave theory are chosen to model the traffic dynamic under a reference timing plan using a phase swap control strategy. The furthest points that the queues of the sorting area and upstream area can reach can be calculated using the proposed models. Because the proposed models consider the stochastic properties of traffic demands, the improved geometric design parameters can reach a balance point between the desire of preventing spillbacks and improving operation efficiency. The proposed method is evaluated through a simulation using the VISSIM SCAPI. The following conclusions were reached:

1. When modeling the traffic dynamics of the upstream area and the sorting area of the pre-signal system, the traffic in the upstream area has more stochastic properties. The furthest point that the queue in the sorting area can reach will be longer the maximum queue, which means the length of sorting area should be longer than it. We use queue theory and shockwave theory, respectively, to locate the furthest points that the queue can reach in both the upstream area and sorting area. The appropriate geometric design can be calculated by considering the boundary conditions and other driving behaviors.
2. The proposed geometric design will be longer than the BMILP model, and shorter than the method using the maximum flow rate. If the storage capacity is not large enough, there is a high probability of queue spillback. Otherwise, the spatial utilization of the road will drop considerably. The proposed method can increase the spatial utilization of the pre-signal system while maintaining a similar delay, queue length, and ratio of flow to saturation flow. The maximum increase in spatial utilization under congested conditions is 7.5%.
3. Though the geometric design and signal timing are collaboratively designed, the reserve capacity for some phase stages is still high. This is mainly because of the limitation of road capacity. We will continue to discuss the way to use geometric design method, such as the reversible lane, to improve the capacity of the intersection with the pre-signal system.

## Supporting information

S1 File Available traffic data used in this study. (XLS)

## References

[1] Papageorgiou M, Diakaki C, Dinopoulou V, Kotsialos A, Yibing W. Review of road traffic control strategies. Proceedings of the IEEE. 2003; 91(12): 2043–2067.

[2] Li Y, Zhao Z, Li P, Huang S, Chen K. Review of traffic signal control methods under over-saturated conditions. Journal of Traffic & Transportation Engineering. 2013; 13(4): 116–126.

[3] Zhao J, Liu Y, Wang T. Increasing signalized intersection capacity with unconventional use of special width approach lanes: special width approach lanes. Computer-Aided Civil and Infrastructure Engineering. 2016; 31(10): 794–810.

[4] Esawey ME, Sayed T. Comparison of two unconventional intersection schemes: crossover displaced left-turn and upstream signalized crossover intersections. Transportation Research Record. 2007; 2023: 10–19.

[5] Xuan Y, Daganzo CF, Cassidy MJ. Increasing the capacity of signalized intersections with separate left turn phases. Transportation Research Part B: Methodological. 2011; 45(5): 769–781.

[6] Zhou Y, Zhuang H. The optimization of lane assignment and signal timing at the tandem intersection with pre-signal. Journal of Advanced Transportation. 2014; 48(4): 362–376.

[7] Yan C, Jiang H, Xie S. Capacity optimization of an isolated intersection under the phase swap sorting strategy. Transportation Research Part B: Methodological. 2014; 60: 85–106.

[8] Cai Z, Xiong M, Ma D, Wang D. Traffic design and signal timing of staggered intersections based on a sorting strategy. Advances in Mechanical Engineering. 2016; 8(4): 1–9.

[9] He H, Guler SI, Menendez M. Adaptive control algorithm to provide bus priority with a pre-signal. Transportation Research Part C: Emerging Technologies. 2016; 64: 28–44.

[10] Wolshon B, Anurag P. Traffic engineering handbook (7^th^ ed). John Wiley & Sons, 2016.

[11] Guler SI, Gayah VV, Menendez M. Bus priority at signalized intersections with single-lane approaches: A novel pre-signal strategy. Transportation Research Part C: Emerging Technologies. 2016; 63: 51–70.

[12] Wu J, Hounsell N. Bus priority using pre-signals. Transportation Research Part A: Policy and Practice. 1998; 32 (8): 563–583.

[13] Guler SI, Menendez M. Analytical formulation and empirical evaluation of pre-signals. Transportation Research Part B: Methodological. 2014; 64: 41–53.

[14] Zhao J, Li P, Zheng Z, Han Y. Analysis of saturation flow rate at tandem intersections using field data. IET Intelligent Transport Systems. 2018; 12(5): 394–403.

[15] Ma W, Xie H, Liu Y, Head L, Luo Z. Coordinated optimization of signal timings for intersection approach with presignals. Transportation Research Record. 2013; 2355: 93–104.

[16] Yang Q, Shi Z. Performance analysis of the phase swap sorting strategy for an isolated intersection. Transportation Research Part C: Emerging Technologies. 2017; 77: 366–388.

[17] Wong CK, Heydecker BG. Optimal allocation of turns to lanes at an isolated signal-controlled junction. Transportation Research Part B: Methodological. 2011; 45 (4): 667–681.

[18] Wong CK, Wong SC. Lane-based optimization of signal timings for isolated junctions. Transportation Research Part B: Methodological. 2003; 37 (1): 63–84.

[19] Bie Y, Liu Z, Wang Y. A real-time traffic control method for the intersection with pre-signals under the phase swap sorting strategy. Plos One. 2017; 12(5):e0177637 10.1371/journal.pone.0177637 28531198 PMC5439707

[20] Zhao J, Liu Y. Safety evaluation of intersections with dynamic use of exit-lanes for left-turn using field data. Accident Analysis & Prevention. 2017; 102:31–40. 28259022 10.1016/j.aap.2017.02.023

[21] Kuang Y, Qu X, Weng J, Etemad-Shahidi A. How does the driver's perception reaction time affect the performances of crash surrogate measures? PloS One. 2015; 10(9): e0138617 10.1371/journal.pone.0138617 26398416 PMC4580564

[22] Autey J, Sayed T, El Esawey M. Operational performance comparison of four unconventional intersection designs using micro-simulation. Journal of Advanced Transportation. 2013; 47(5): 536–552.

[23] El Esawey M, Sayed T. Analysis of unconventional arterial intersection designs (UAIDs): state-of-the-art methodologies and future research directions. Transportmetrica A: Transport Science. 2013; 9(10): 860–895.

[24] Wu J, Liu P, Tian ZZ, Xu C. Operational analysis of the contraflow left-turn lane design at signalized intersections in China Transportation Research Part C: Emerging Technologies. 2016; 69: 228–241.

[25] Li Y, Li K, Tao S, Wan X, Chen K. Optimization of the design of pre-signal system using improved cellular automaton. Computational Intelligence & Neuroscience. 2014; 926371: 1–11. 10.1155/2014/926371 PMC4236971 25435871

[26] Federal Highway Administration. Manual on Uniform Traffic Control Devices for Streets and Highways. Datamotion Publishing LLC 2013.

[27] Winston W. Operations research: applications and algorithms (4th ed). Cengage Learning, 2003.

[28] Li Y, Guo X, Yang J, Liu Y, He S. Mechanism analysis and implementation framework for traffic signal control of over-saturated intersection group. Journal of Transportation Systems Engineering & Information Technology. 2011; 11(4): 28–34.

[29] Siam MRK, Nasrin S, Hadiuzzaman M, Muniruzzaman SM, Haque N. VISCAL: Heuristic algorithm based application tool to calibrate microscopic simulation parameters. Journal of Traffic & Transportation Engineering(English Edition). 2017; 5(1): 28–43.

